# Thymidine Phosphorylase/β-tubulin III expressions predict the response in Chinese advanced gastric cancer patients receiving first-line capecitabine plus paclitaxel

**DOI:** 10.1186/1471-2407-11-177

**Published:** 2011-05-18

**Authors:** Jing Gao, Ming Lu, Jing-wei Yu, Yan-yan Li, Lin Shen

**Affiliations:** 1 Key laboratory of Carcinogenesis and Translational Research (Ministry of Education), Department of GI Oncology, Peking University School of Oncology, Beijing Cancer Hospital & Institute, Beijing, China

## Abstract

**Background:**

To assess the role of Thymidine Phosphorylase and β-tubulin III in clinical outcome of Chinese advanced gastric cancer patients receiving first-line capecitabine plus paclitaxel.

**Methods:**

The clinical data and tumor biopsies prior treatment from 33 advanced gastric cancer patients receiving capecitabine plus paclitaxel (cohort 1, experimental group) and 18 patients receiving capecitabine plus cisplatin (cohort 2, control group) in Beijing Cancer Hospital from July 2003 to December 2008 were retrospectively collected and analyzed for Thymidine Phosphorylase and β-tubulin III expressions by immunohistochemistry. The relationships between expressions of biomarkers and response or survival were determined by statistical analysis.

**Results:**

The median age of 51 patients was 57 years (range, 27-75) with male 34 and female 17, and the response rate, median progression-free survival and overall survival were 43.1%, 120d and 265d. Among cohort 1, the response rate, median progression-free survival and overall survival in β-tubulin III positive (n = 22) and negative patients (n = 11) were 36.4%/72.7% (positive vs negative, *P *= 0.049), 86d/237d (*P *= 0.046) and 201d/388d (*P *= 0.029), respectively; the response rate (87.5% vs 14.3%, *P *= 0.01) and median progression-free survival (251d vs 84d, *P *= 0.003) in Thymidine Phosphorylase positive & β-tubulin III negative patients (n = 8) were also significantly higher than those in Thymidine Phosphorylase negative & β-tubulin III positive patients (n = 7). There was no correlation between β-tubulin III expression and response or survival among cohort 2 (n = 18).

**Conclusions:**

In Chinese advanced gastric cancer, Thymidine Phosphorylase positive & β-tubulin III negative might predict response and prognosis to capecitabine plus paclitaxel chemotherapy. Further prospective evaluation in large samples should be performed to confirm these preliminary findings.

## Background

Gastric cancer remains one of the most common causes of cancer death worldwide [1], especially in China [2]. Although the improvement of diagnostic methods enables some patients to receive radical cure at early disease, about 40% patients still miss the opportunity of radical cure at the time of diagnosis, furthermore, about 50% patients occur relapse and metastasis after operation. For these patients, chemotherapy is still the main method general accepted in the world. Several agents are now available for the systemic chemotherapy of patients with gastric cancer, including fluoropyrimidines, platinum, taxanes and so on. But fluoropyrimidines are fundamental in gastric cancer. For advanced gastric cancer patients (AGC), combination chemotherapy with two or three drugs is most common with superiority compared to best supportive care in first-line or second-line therapy [3-6].

Capecitabine is an orally-administered chemotherapeutic agent which was designed to generate 5-Fluorouracil (5-FU) preferentially in tumors. It is a prodrug which is converted to 5-FU in the tumors through a pathway with three enzymatic steps and two intermediary metabolites involved in. At the last enzymatic step, the metabolite 5'-deoxy-5-fluorouridine (5'-DFUR) is converted to 5-FU by Thymidine Phosphorylase (TP) which is more active in tumor tissues than in normal tissues [7]. So, the overexpression of TP in tumor tissues can increase the concentration of 5-FU and thus enhance the anticancer effect. Current evidences indicate that the expression level of TP may influence the clinical outcome of capecitabine in non-small cell lung cancer, gastrointestinal adenocarcinoma, breast cancer, head and neck cancer, and so on [8-11]. Two large phase III clinical trials showed that capecitabine could substitute 5-FU in clinical administration [12,13]. Also capecitabine combined with cisplatin or paclitaxel has been proved to be an effective combination regimen used in patients with advanced gastric cancer as first-line or second-line treatment [13-15].

Taxane including paclitaxel (PTX) and docetaxel, is another active antitumor agent for gastric cancer. Taxane binds to β-tubulin, which is one of the major components of microtubule, and exerts its growth-inhibitory effects through the stabilization of microtubule, resulting in the growth arrest of tumor cells at the G2/M phase [16]. Several mechanisms have been suggested as responsible for taxane resistance: ① The overexpression of *MDR1 *gene which encodes P-glycoprotein able to efflux taxanes and other cationic drugs, thereby hampering drug retention [17]; ② Point mutation in tubulin has been identified to be responsible for taxane resistance [18,19]. ③ The selective overexpression of β-tubulin isotypes is another mechanism of resistance [20]. Up to now, at least seven distinct β-tubulin isotypes (classes I, II, III, IVa, IVb, V and VI) have been reported in human, with a complex distribution pattern in various tissues [20]. Some researches found the presence of class III β-tubulin (β-tubulin III, TUBB3) inhibited the assembly of β-tubulin subunits promoted by paclitaxel [21] and TUBB3 expressed in some paclitaxel-resistant cells [22]. Many preclinical studies have shown high expression levels of TUBB3 are associated with paclitaxel resistance in human lung cancer [23], ovarian cancer [22], prostate cancer [24] and breast cancer [25] cell lines. In studies of lung cancer, breast cancer and ovarian cancer, there are reverse relations between TUBB3 expression and paclitaxel efficacy or prognosis of patients [26-28].

Now that the target of paclitaxel is β-tubulin, and study had reported that there were rare mutations in β-tubulin for gastric cancer (no mutations were found in 50 tumor samples) [29], we considered that the overexpression of TUBB3 was the most probable mechanism of paclitaxel resistance in gastric cancer. This study was designed to demonstrate the clinical implications of TP and TUBB3 expressions in capecitabine plus paclitaxel chemotherapy for advanced gastric cancer patients, and to identify potential predictors for patients with gastric cancer treated with capecitabine plus paclitaxel.

## Methods

### Patients Eligibility

All patients in this study were retrospectively collected as following criteria: patients had histologically confirmed metastatic gastric adenocarcinoma and at least one measurable lesion according to the response evaluation criteria in solid tumors guidelines [30]; patients were treated by capecitabine plus paclitaxel or cisplatin in gastrointestinal department of Beijing Cancer Hospital from July 2003 to December 2008, and had completed at least two cycles of chemotherapy; no any chemotherapy except for neoadjuvant or adjuvant chemotherapy (adjuvant chemotherapy completed over 12 months) was done; all patients underwent endoscopic biopsy from primary stomach before chemotherapy.

### Treatment Regimens

The first-line chemotherapy regimens with capecitabine plus paclitaxel or cisplatin were administered to patients as following: capecitabine (Roche Laboratories Inc., Nutley, NJ) was given orally at a dose of 1,250 mg/m^2 ^twice daily from day1 (d1) to day14 (d14) of 3-weeks cycle; paclitaxel (Hainanhaiyao Co., Ltd., China) was given at a dose of 80 mg/m^2 ^by a 180-min i.v. infusion on d1 and d8 of each cycle; cisplatin (Qilu Pharmaceutical CO., LTD., China) was given at a dose of 80 mg/m^2 ^by a 240-min i.v. infusion on d1 of each cycle. Treatment was continued until disease progression or unacceptable toxicity, or patients/physicians' decision.

### Response Evaluation

Chemotherapeutic response was evaluated every two months by computed tomography (CT) according to the Response Evaluation Criteria in Solid Tumors (RECIST) criteria. Patients were categorized by complete response (CR), partial response (PR), stable disease (SD), and progressive disease (PD). CR and PR patients were defined as responders, SD and PD patients as nonresponders. The progression-free survival (PFS) and overall survival (OS) were calculated from the first day of therapy to disease progression and death from any cause, respectively.

### Immunohistochemistry analysis for TP and TUBB3

All tumor samples were retrospectively collected from patients, and two step method of immunohistochemistry (IHC) was used to detect TP and TUBB3 in tumor sections. Formalin-Fixed Paraffin-Embedded tissue sections with 4 μm thick were deparaffinized in xylene and hydrated in graded alcohols. After antigen retrieval in 0.01M citrate buffer (pH 6.0), sections were treated with endogenous peroxidase confining liquid (Beijing CoWin Biotech Co., Ltd., Lot. CW0117) for 10 min. Sections were rinsed and incubated with TUBB3 and TP (Beijing CoWin Biotech Co., Ltd.) monoclonal antibodies for 60 min, respectively. After rinsing in phosphate buffered saline (PBS), the sections were incubated with general type IgG-HRP Polymer (Beijing CoWin Biotech Co., Ltd., Lot. CW0117) for 10 min, followed by chromogenic 3,3'-Diaminobenzidine tetrahydrochloride dihydrate (DAB) for about 2-7min. Finally, sections were conterstained with hematoxylin for 1 min followed by dehydrated in graded alcohols, cleared in xylene, and covered with coverslips. Each experiment included negative control. Sections were examined and scored by two independent professional pathologists of pathology department without any knowledge of this study. TP protein was distributed in cytoplasm and nuclear, TUBB3 in cytoplasm. Staining was graded for intensity of staining according to previous description [31]. Briefly, intensity of staining (1, weak; 2, moderate; 3, strong) and percentage of cells stained (1, 0%~10%; 2, 11%~50%; 3, 51%~100%) were calculated. At last, the expression levels were considered to be positive or negative based on the median staining score (intensity score plus percentage score) as following: negative for score ≤ 3, positive for score > 3.

### Statistical Analysis

According to TP and TUBB3 protein expression levels, patients were divided into two groups (positive and negative). The relationships between TP, TUBB3 expressions and response to capecitabine plus paclitaxel or cisplatin were analyzed using Fisher's exact test. Kaplan-Meier curves and log-rank test were used to analyze the association between expression levels of biomarkers and survival. Statistical analysis was done using SPSS 13.0 (SPSS Inc, Chicago, Illinois, USA).

## Results

### Patient Demographics

Fifty-one patients were included in this study between July 2003 to December 2008 in our hospital with male 34, female 17, median age 57 years (range 27-75 years). Thirty-three patients (male 20, female 13, median age 57 years [range 27-74 years]) received capecitabine plus paclitaxel with a median 6 cycles of chemotherapy (cohort 1) and eighteen patients (male 14, female 4, median age 57 years [range 42-75 years]) received capecitabine plus cisplatin with a median 6 cycles of chemotherapy (cohort 2). The characteristics of 51 patients are presented in Table 1.

**Table 1:** Patient Demographics and Clinical Characteristics

Characteristic	Cohort 1 (n = 33)		Cohort 2 (n = 18)		Total (n = 51)	
	
	No. of Patients	%	No. of Patients	%	No. of Patients	%
**Sex**						
Male	20	60.6	14	77.8	34	66.7
female	13	39.4	4	22.2	17	33.3
**Age, years**						
Median	57		57		57	
Range	27-74		42-75		27-75	
**KPS**						
90-100	23	69.7	14	77.8	37	72.5
70-80	10	30.3	4	22.2	14	27.5
**Sites of metastatic disease**						
Liver	12	36.4	6	33.3	18	35.3
Lung	1	3.0	4	22.2	5	9.8
Lymph nodes	33	100	17	94.4	50	98.0
Peritoneum	6	18.2	3	16.7	9	17.6
Others*	8	24.2	4	22.2	12	23.5
**Histological differentiation**^**#**^						
Poor	25	75.8	11	61.1	36	70.6
Good	8	24.2	7	38.9	15	29.4

NOTE: *Including ovary, adrenal glands, subcutaneous of abdominal wall, pelvic cavity, bone and thoracic cavity. ^#^Poor, including poorly differentiated adenocarcinoma, signet ring cell carcinoma and mucinous adenocarcinoma; Good, including moderate-well differentiated adenocarcinoma. KPS, Kamofsky performance status.

### Response Evaluation and Survival

Up to February 2010, all patients had been evaluable for response and 43 patients died. The overall response rate (CR+PR) in 51 patients was 43.1%, with 22 partial responders (43.1%), 18 patients with stable disease (35.3%), and 11 patients with progressive disease (21.6%). The median PFS and OS of 51 patients were 120 days and 265 days, respectively. There were no significant differences of response and survival between cohort 1 and cohort 2 (Table 2). The response rate, median PFS and OS in cohort 1 and cohort 2 were 48.5%, 120 days, 252 days and 33.3%, 116 days, 265 days, respectively.

**Table 2:** Response and Survival for all patients

	Cohort 1 (n = 33)		Cohort 2 (n = 18)		*P*	Total (n = 51)	
			
Outcome	**No**.	%	**No**.	%		**No**.	%
CR+PR No.	16	48.5	6	33.3		22	43.1
SD No.	11	33.3	7	38.9		18	35.3
PD No.	6	18.2	5	27.8		11	21.6
Response rate	16	48.5	6	33.3	0.668	22	43.1
Median PFS (days)	120		116			120	
95% CI	77.2-162. 8		0-267.1		0.377	79.7-160.3	
Median OS (days)	252		265			265	
95% CI	157. 5-346.5		107.0-423.0		0.354	181.0-349.0	

NOTE: PFS, progression-free survival; OS, overall survival; 95% CI: 95% confidence interval

### TP IHC and Response, Survival

**Figure 1:**
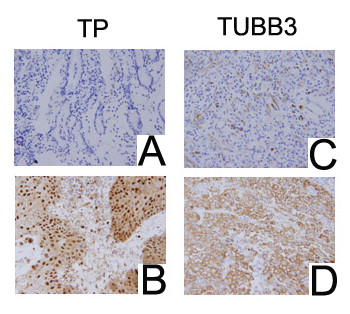
**IHC presenting TP and TUBB3 expressions (magnification, ×400). A, TP negative; B, TP positive; C, TUBB3 negative; D, TUBB3 positive**.

Negative and positive staining for TP protein in 51 tumor samples were 26 and 25 samples (Figure 1, left lane). Of the 25 patients with positive TP, there were 14 responses (56%), compared with 8 of 26 responses (30.8%) seen in negative TP tumors (*P *= 0.069, Table 3). Also, the median PFS and OS in TP positive samples were longer than that in TP negative samples, but significant difference only existed in OS (*P *= 0.017) not in PFS (*P *= 0.613) between two groups (Table 3).

**Table 3:** Association Between TP Expression and Response or Survival

	TP Staining (n = 51)	
	
	Positive	Negative
**CR+PR No**.	14	8
**SD No**.	5	13
**PD No**.	6	5
**Response rate (%)**	56	30.8
***P***	0.069	
**Median PFS (days)** **(95% CI)**	156 (71.5-240.5)	107 (66.7-147.3)
***P***	0.613	
**Median OS (days)** **(95% CI)**	365 (123.0-567.0)	214 (74.8-317.2)
***P***	0.017	

### TUBB3 IHC and Response, survival

**Figure 2:**
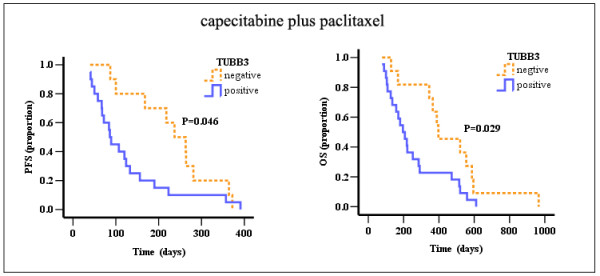
**PFS and OS curves for TUBB3 negative and positive patients receiving capecitabine plus paclitaxel**.

**Figure 3:**
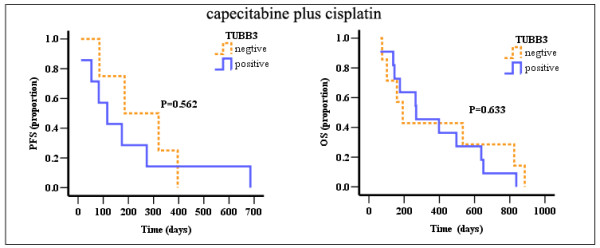
**PFS and OS curves for TUBB3 negative and positive patients receiving capecitabine plus cisplatin**.

IHC of TUBB3 was done in cohort 1 and cohort 2, negative and positive staining for TUBB3 in cohort 1 (33 samples) were 11 and 22 samples (Figure 1, right lane). Among cohort 1, of the 22 patients with positive TUBB3, there were 8 responses (36.4%), compared with 8 of 11 responses (72.7%) seen in negative TUBB3 tumors (*P *= 0.049); also, the median PFS (*P *= 0.046) and OS (*P *= 0.029) in TUBB3 positive samples were much shorter than those in TUBB3 negative samples (Table 4 and Figure 2). Among cohort 2, the response rates in TUBB3 negative patients (n = 7) and positive patients (n = 11) were 28.6% and 36.4%, respectively (*P *= 0.73). Also, there weren't correlations between TUBB3 expressions and median PFS (*P *= 0.562) or OS (*P *= 0.633) in cohort 2 (Figure 3).

**Table 4:** Association Between TUBB3 Expression and Response or Survival

	TUBB3 Staining	
	
	Positive	Negative
**CR+PR No**.	8	8
**SD No**.	9	2
**PD No**.	5	1
**Response rate (%)**	36.4	72.7
***P***	0.049	
**Median PFS (days)** **(95% CI)**	86 (40.9-131.1)	237 (19.4-454.6)
***P***	0.046	
**Median OS (days)** **(95% CI)**	201 (182.5-253.5)	388 (67.6-708.4)
***P***	0.029	

### TP, TUBB3 IHC and Response, survival

**Figure 4:**
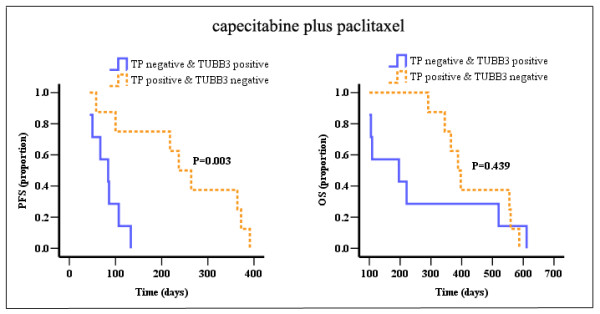
**PFS and OS curves for TP negative & TUBB3 positive and TP positive & TUBB3 negative patients receiving capecitabine plus paclitaxel**.

Among cohort 1, TP positive & TUBB3 negative staining was 8 samples, with 7 responses (87.5%), but for TP negative & TUBB3 positive staining samples, only 1 of 7 responses (14.3%) (*P *= 0.01, Table 5). The median PFS (251d) and OS (393d) in TP positive & TUBB3 negative staining samples were longer than those (PFS: 84d, OS: 196d) in TP negative & TUBB3 positive staining samples, although there was no statistical difference of OS between two groups (*P *= 0.003 for PFS, *P *= 0.439 for OS, Table 5 and Figure 4). There were 15 samples (8 responses, 53.3%) displaying TP positive & TUBB3 positive staining with median PFS 122d and median OS 207d, and 3 samples (all with SD) displaying TP negative & TUBB3 negative staining.

**Table 5:** Association Between TP, TUBB3 Expressions and Response or Survival

	TP, TUBB3 Staining	
	
	TP positive & TUBB3 negative	TP negative & TUBB3 positive
**CR+PR No**.	7	1
**SD No**.	1	3
**PD No**.	0	3
**Response rate (%)**	87.5	14.3
***P***	0.01	
**Median PFS (days)** **(95% CI)**	251 (146.4-354.6)	84 (52.5-110.7)
***P***	0.003	
**Median OS (days)** **(95% CI)**	393 (340.9-530.8)	196 (70.3-461.7)
***P***	0.439	

## Discussion

The treatment of gastric cancer is still a troublesome problem worldwide, and up to now there is not systematic standard regimen for gastric cancer. Over 50% patients couldn't respond to chemotherapy with the major obstacle is drug resistance. So how to improve the response and extend the life of patients is requested urgently. At present, the regimen of platinum (cisplatin, oxaliplatin, etc.) combined with fluorouracil (5-FU, capecitabine, etc.) is most used for gastric cancer in China. Capecitabine is a new type flurouracil carbamates antitumor agent and widespread used by tumor patients due to its convenient administration, well tolerance, definite effect and low side effect. Animal experiments have shown that many drugs, such as irinotecan, oxaliplatin, cisplatin and paclitaxel, had synergistic effect with capecitabine [32-34], and capecitabine plus paclitaxel or cisplatin regimens have been considered as desirable chemotherapy in clinical studies [13-15].

Based on our results, the response rate of capecitabine plus paclitaxel or cisplatin was 48.5% and 33.3%, respectively. However, although these regimens were effective in gastric cancer, over an half patients failed to respond due to drug resistance. So if we can find some predictive biomarkers for capecitabine plus paclitaxel to guide treatment of patients, there must be a very great improvement for response and survival. TP is a key enzyme in the metabolic pathway of capecitabine. TP enzyme, also called tumor related angiogenesis factor, is higher expressed in tumor tissues than in normal tissues, then the concentration of 5-FU in tumor tissues is raised followed by enhanced antitumor activity. In our results, the response rate, PFS and OS for TP positive patients are all higher than that in TP negative patients, which is similar with the results by other researchers [8-11]. According to above results, we analyzed TP expression in advanced gastric cancer and found the overall survival of TP positive patients was much better than that in TP negative patients. However, in different studies of colorectal cancer, data about the prognostic or predictive value of TP were conflicting: Meropol NJ et al [35] reported TP expression might be a predictive marker for capecitabine response, but Koopman et al [36] found TP expression didn't show a predictive or prognostic value for capecitabine combination chemotherapy.

Taxanes are a kind of antitumor drugs and mechanisms about its resistance have been studied for a long time. Microtubule is the target of paclitaxel which induces microtubule stabilization, inhibits microtubule dynamics and interrupts cell divisions. Studies showed that TUBB3 high expressed in paclitaxel-resistant cells [37] and after transfecting TUBB3 cDNA into mammalian cells, cells with TUBB3 expression displayed resistant to paclitaxel [38]. Also in other studies about breast cancer, ovary cancer, head and neck neoplasms, there were relationship between TUBB3 expression and response or survival of paclitaxel. We studied the correlation in 33 samples treated with capecitabine plus paclitaxel, and found that the response rate for TUBB3 negative patients was 72.7%, but only 36.4% in TUBB3 positive patients. Moreover, in cohort 1, the prognosis of TUBB3 negative patients was much better than that of TUBB3 positive patients. Our results indicated there was not relationship between TUBB3 expression and response or survival in patients receiving capecitabine plus cisplatin, so TUBB3 expression could act as a predictor of paclitaxel efficacy.

TP could be upregulated after the treatment of taxane in preclinical trial [39], we analyzed the relationship between TP, TUBB3 expressions and the response or survival of patients. Interestingly, the response rate in TP positive & TUBB3 negative patients was 87.5%, but only 14.3% in TP negative & TUBB3 positive patients. This result will need to be further confirmed in future studies.

Recently, many studies put the sights into genomic polymorphisms in genes involved in drug metabolic pathway and correlated with the target of agents. Few genes have been used to guide clinical medication, such as K-RAS [40], C-KIT [41], EGFR [42], Her-2 [43], and so on, but for most drugs there were no predictive markers. According to our results, TP and TUBB3 may be prospective to be used to predict the response and survival of capecitabine and paclitaxel.

To summarize our results, our findings demonstrate it's possible that overexpression of TUBB3 is the major reason of paclitaxel resistance in gastric cancer, and positive TP & negative TUBB3 expressions might predict response and prognosis to capecitabine plus paclitaxel chemotherapy in AGC.

## Conclusions

Our findings suggested that, in Chinese advanced gastric cancer, TP positive & TUBB3 negative expressions might predict response and prognosis to capecitabine plus paclitaxel chemotherapy. Further prospective evaluation in large samples should be performed to confirm these preliminary results.

## Declaration of competing interests

The authors declare that they have no competing interests.

## Authors' contributions

Jing Gao and Ming Lu contributed equally to this work; Jing Gao, Ming Lu and Yan-yan Li performed the experiments; Jing Gao wrote the manuscript; Jing-wei Yu performed the statistical analysis; Lin Shen designed the experiments and revised the manuscript. All authors have read and approved the final manuscript.

## Pre-publication history

The pre-publication history for this paper can be accessed here:

http://www.biomedcentral.com/1471-2407/11/177/prepub
